# Clinical Effectiveness and Accuracy of Fully Digital Workflows Versus Conventional Methods in Implant-Supported Restorations: A Systematic Review and Meta-Analysis

**DOI:** 10.7759/cureus.102864

**Published:** 2026-02-02

**Authors:** Abdulaziz Zailai, Nora F Aldwyyan, Marwan H Alsaedy, Amani Alasiri, Reema K Alharbi, Ayman R Alothman, Ali S Metwaly

**Affiliations:** 1 Restorative Dentistry, Jazan Specialized Dental Center, Jazan, SAU; 2 Restorative Dentistry, Scientific Council of the Saudi Board in Restorative Dentistry (SBRD) in the Southern Region, Jazan, SAU; 3 General Dentistry, Saudi Arabia Ministry of Health (MOH), Riyadh, SAU; 4 General Department of Medical Services, Ministry of Interior (MOI), Riyadh, SAU; 5 Dentistry, Saudi Arabia Ministry of Health (MOH), Riyadh, SAU; 6 Dentistry, Vision College, Riyadh, SAU; 7 Oral and Maxillofacial Surgery, King Saud Medical City, Riyadh, SAU; 8 Medicinal Chemistry and Drug Discovery, Faculty of Pharmacy, Alexandria University, Alexandria, EGY

**Keywords:** clinical accuracy, conventional impression, digital dentistry, implant-supported restorations, intraoral scanning, meta-analysis, patient-reported outcome measures, systematic review, time efficiency

## Abstract

The transition from conventional analogue techniques to digital workflows is transforming implant dentistry. However, a comprehensive synthesis of comparative clinical evidence is lacking. This systematic review and meta-analysis aimed to evaluate the clinical effectiveness, accuracy, and patient-centred outcomes of fully digital versus conventional workflows for fabricating implant-supported restorations. This review was conducted following Preferred Reporting Items for Systematic reviews and Meta-Analyses (PRISMA) guidelines and prospectively registered with PROSPERO (International Prospective Register of Systematic Reviews). A comprehensive search of PubMed/MEDLINE, Embase, and Cochrane Library was performed to identify randomized controlled trials (RCTs) and non-randomized clinical studies comparing digital (intraoral scanning) and conventional (elastomeric impression) restorative workflows for single-unit or full-arch implant prostheses. Two reviewers independently performed study selection, data extraction, and risk-of-bias assessment using the Cochrane RoB 2 and ROBINS-I tools. The primary outcomes were time efficiency, accuracy/fit, and patient-reported outcome measures (PROMs). Secondary outcomes included biological and prosthetic complication rates. Random-effects meta-analyses were conducted using the Hartung-Knapp method, with heterogeneity assessed using the statistic. The certainty of the evidence was evaluated using the GRADE approach. Ten clinical studies (six RCTs, four non-randomized) met the inclusion criteria. Most RCTs demonstrated a low risk of bias, while non-randomized studies showed a moderate risk of bias. Meta-analysis revealed that digital workflows significantly reduced mean procedure time compared to conventional methods (mean difference (MD): -4.28 minutes; 95% CI: -8.40 to -0.16; *p* < 0.05). No statistically significant difference was found in restorative accuracy or fit between the two workflows (standardized mean difference (SMD): 0.20; 95% CI: -3.72 to 4.12). Patient-reported outcomes showed a trend toward favoring the digital workflow for comfort and satisfaction (MD: 13.98; 95% CI: -7.74 to 35.70). Implant survival rates were high (>98%) and comparable for both groups, with no significant difference in complication rates. The certainty of the evidence ranged from Low for accuracy to Moderate for time efficiency and PROMs. Fully digital workflows for implant-supported restorations are significantly more time-efficient and are preferred by patients, while demonstrating comparable clinical accuracy and survival rates to conventional methods. These findings support the adoption of digital technologies as a robust and effective alternative in modern implant prosthodontics.

## Introduction and background

The integration of digital technologies into prosthodontics has transformed dental rehabilitation, driving a shift from analogue to computer-aided workflows. The digitization of clinical protocols, encompassing intraoral scanning (IOS), computer-aided design (CAD), and computer-aided manufacturing (CAM), promises to enhance procedural efficiency, streamline communication between clinicians and laboratories, and improve the predictability of treatment outcomes [1,2]. While conventional methods that depend on elastomeric impressions and lost-wax casting have long served as the gold standard, they are susceptible to material distortion, expansion of gypsum casts, and operator-dependent errors that may compromise the final restoration [3]. The adoption of digital workflows aims to circumvent these cumulative errors while improving patient comfort and reducing chairside time [4].

In implant dentistry, the accuracy of the prosthetic workflow is crucial. Unlike natural teeth, osseointegrated implants lack the compensatory mechanism of the periodontal ligament, requiring a passive fit of the superstructure to prevent mechanical complications such as screw loosening and biological complications such as marginal bone loss [5,6]. Achieving this level of precision, particularly in extensive rehabilitation, requires rigid splinting of the impression copings and meticulous verification steps. The introduction of IOS systems has challenged these traditional protocols, with recent meta-analyses suggesting that digital impressions may offer superior time efficiency and higher patient satisfaction than conventional techniques [2,5].

Despite the rapid adoption of IOS and digital restorative technologies, the scientific validation of digital workflows for implant-supported restorations depends on preclinical evidence (i.e., in vitro and laboratory-based studies). Numerous in vitro studies have demonstrated that digital impressions can achieve trueness and precision comparable to or even exceeding those of conventional methods for single-unit and short-span restorations [7-9]. However, the accuracy of IOS in full-arch scenarios remains debatable. Laboratory studies indicate that as the scan span increases, the accumulation of stitching errors may result in deviations that exceed clinically acceptable thresholds, particularly when compared with conventional open-tray techniques [10-12]. Furthermore, variables such as implant angulation, inter-implant distance, and geometry of the scan bodies have been shown to significantly influence digital acquisition accuracy in controlled settings [13,14]. While emerging technologies such as photogrammetry aim to mitigate these cross-arch distortions [4], translating these in vitro findings to the clinical environment, characterized by patient movement, saliva, and soft tissue mobility, remains complex.

The clinical efficacy of digital workflows has been documented for tooth-borne restorations [15,16]. Randomized trials comparing digital and conventional workflows for natural teeth have reported high survival rates, high precision in marginal fit, and distinct patient preference for optical scanning [17,18]. Clinical evidence specifically targeting implant-supported restorations is often fragmented. Previous research has focused on surgical placement accuracy [19] or specific proprietary workflows lacking a conventional control [20,21], rather than the restorative outcome itself. Additionally, operator experience and the learning curve associated with digital devices have been identified as confounding factors that may influence clinical success, distinct from the technology’s inherent capabilities [22].

Because of the disparity between in vitro data and the realities of clinical practice, there is a need to critically appraise the performance of fully digital workflows in vivo. Therefore, this systematic review and meta-analysis aimed to evaluate the clinical effectiveness and accuracy of fully digital workflows compared with conventional methods for implant-supported restorations, with a specific focus on passivity of fit, complication rates, time efficiency, and patient-reported outcome measures (PROMs).

## Review

Methods

Protocol and Registration

This systematic review and meta-analysis were conducted in accordance with the Preferred Reporting Items for Systematic Reviews and Meta-Analyses (PRISMA) guidelines [23]. The study protocol was prospectively registered in the International Prospective Register of Systematic Reviews (PROSPERO; CRD420251274514).

Search Strategy

A literature search was performed in the electronic databases PubMed/MEDLINE, Embase, and Cochrane Library. The search strategy combined controlled vocabulary (MeSH terms) and free-text keywords relevant to "digital workflow", "intraoral scanning", "conventional impression", and "implant-supported restorations". The search was supplemented by manually screening the reference lists of the included studies and relevant systematic reviews to identify additional eligible articles. No time restrictions were applied; however, only studies published in English were considered for inclusion.

Inclusion Criteria

Studies were eligible for inclusion if they were randomized controlled trials (RCTs) or non-randomized prospective/retrospective clinical studies; involved human participants requiring implant-supported restorations (single crowns or full-arch); compared a fully digital workflow (intraoral scanning) with a conventional workflow (elastomeric impressions); and reported quantitative data on clinical outcomes such as passivity of fit, marginal adaptation, time efficiency, complication rates, or PROMs. In vitro studies, case reports, animal studies, and reviews were also excluded.

Study Selection and Data Extraction

Two independent reviewers screened the titles and abstracts for eligibility, followed by a full-text assessment of potentially relevant articles. Data extraction was performed using a standardized form to collect information on the study design, participant demographics, intervention details, and outcome measures. The inter-rater reliability for study selection and data extraction was assessed using Cohen’s Kappa statistic to quantify the level of agreement between reviewers [24]. Any discrepancies were resolved through discussion or consultation with a third reviewer.

Risk-of-Bias Assessment

The methodological quality of the included studies was evaluated using study-design-specific tools. For RCTs, the risk of bias was assessed using the Cochrane risk of bias 2 (RoB 2) tool [25], covering domains such as the randomization process, deviations from intended interventions, missing outcome data, measurement of the outcome, and selection of the reported result. For non-randomized studies of interventions, the Risk Of Bias In Non-randomized Studies of Interventions (ROBINS-I) tool was employed to evaluate bias across pre-intervention, at-intervention, and post-intervention domains [26].

Statistical Analysis

All statistical analyses were performed using R statistical software (version 4.5.1, R Foundation for Statistical Computing, Vienna, Austria, https://www.R-project.org/) [27,28].

Effect Measures and Synthesis

Outcomes were synthesized using appropriate effect measures: mean difference (MD) or standardized mean difference (SMD) for continuous variables (e.g., time efficiency, fit accuracy) and risk ratio (RR) for dichotomous variables (e.g., complication rates, survival). A random-effects model was used for all meta-analyses to account for the anticipated clinical and methodological variability across studies [29]. The Hartung-Knapp-Sidik-Jonkman adjustment was applied to provide a more robust variance estimation, particularly given the expected small number of included studies [30]. Results were presented with 95% confidence intervals (CIs) and prediction intervals to estimate the potential range of effects in future clinical settings [31].

Heterogeneity and Robustness

Statistical heterogeneity was quantified using the I^2^ statistic and χ2 test for inconsistency [32]. The between-study variance was further assessed using Tau-squared (τ2) [33]. To explore the potential sources of heterogeneity, subgroup analyses and meta-regression were conducted based on moderators such as clinical indication (single crown vs. full-arch) and workflow type. Sensitivity analyses were performed to test the robustness of the results by excluding studies with a high risk of bias or those contributing significantly to heterogeneity.

Assessment of Reporting Bias

Publication bias and small study effects were visually assessed using funnel plots [34]. Statistical asymmetry was formally tested using Egger’s regression [35] and Begg’s rank correlation [36] tests. Reporting and dissemination biases were qualitatively evaluated by comparing published outcomes with study protocols, where available.

Certainty of Evidence

The overall certainty of the evidence for each outcome was graded using the Grading of Recommendations Assessment, Development, and Evaluation (GRADE) approach [37]. Evidence was categorized as high, moderate, low, or very low based on considerations of risk of bias, inconsistency, indirectness, imprecision, and publication bias.

Results

Study Selection and Characteristics

**Figure 1:**
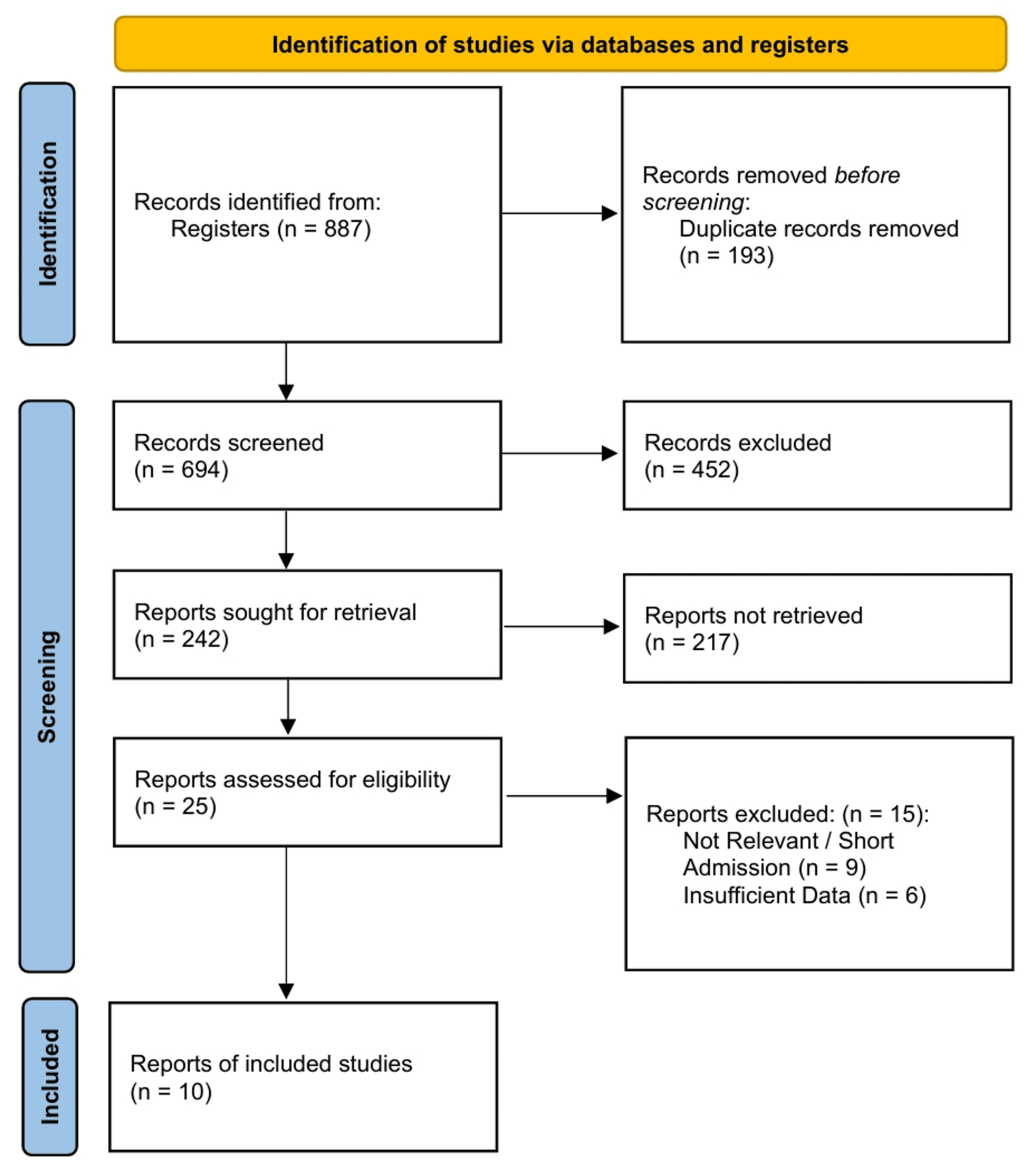
PRISMA 2020 flow diagram.

The literature search yielded 887 initial records. After deduplication and rigorous screening against inclusion criteria, 10 primary clinical studies were included in the final qualitative and quantitative syntheses [38-47]. The selection process is illustrated in the PRISMA flowchart (Figure 1).

The included studies comprised six RCTs [38,39,41,44,46,47], one non-randomized comparative clinical study [43], and three prospective/retrospective clinical studies [40,42,45]. Publication dates ranged from 2015 to 2024, reflecting the rapid evolution of digital implant workflows (Table 1).

**Table 1:** Characteristics of the included studies. Abbreviations: FIPS, Functional Implant Prosthodontic Score; IOS, intraoral scanner; MBL, marginal bone loss; N, number of patients; PROMs, patient-reported outcome measures; PVS, polyvinylsiloxane; RCT, randomized controlled trial

Study ID	Design	Country	N	Indication	Follow-up	Intervention (digital)	Comparison (conventional)	Primary outcomes measured
Beck et al. [38]	RCT	Austria	27	Single crown	4 years	TRIOS 3 (IOS)	Polyether impression	MBL, complications, PROMs, FIPS
Capparè et al. [44]	RCT	Italy	50	Full arch (immediate load)	4 years	Carestream CS 3600 (IOS)	Gypsum impression	Time, MBL, survival, complications
Chochlidakis et al. [42]	Prospective	USA	16	Full arch	Cross-sectional	True Definition (IOS)	PVS impression (open-tray)	3D accuracy
Corsalini et al. [39]	RCT	Italy	72	Single crown	1 year	TRIOS 3 (IOS)	PVS impression	Fit, time, comfort (PROMs)
De Angelis et al. [40]	Retrospective	Italy	150	Full arch (immediate load)	2 years	TRIOS 3 Shape (IOS)	PVS impression (splinted)	Fit, survival, MBL, satisfaction
Delize et al. [45]	RCT (Intrasubject)	Belgium	31	Single crown	Cross-sectional	TRIOS (IOS)	Silicone impression	Fit, PROMs, aesthetics
Gedrimiene et al. [43]	Comparative Clinical	Lithuania	6	2-4-unit bridge	Cross-sectional	TRIOS 3 (IOS)	PVS impression (splinted)	3D accuracy (mismatch)
Hashemi et al. [41]	RCT (Crossover)	Iran	10	3-unit bridge	6 months	TRIOS 3 (IOS)	PVS impression	Accuracy, time, passivity, aesthetics
Joda & Brägger [46]	RCT	Switzerland	20	Single crown	Cross-sectional	iTero (IOS)	Polyether impression	Time, costs
Joda & Brägger [47]	RCT (Crossover)	Switzerland	20	Single crown	Cross-sectional	iTero (IOS)	Polyether impression	PROMs, time

Risk-of-Bias Assessment

**Figure 2:**
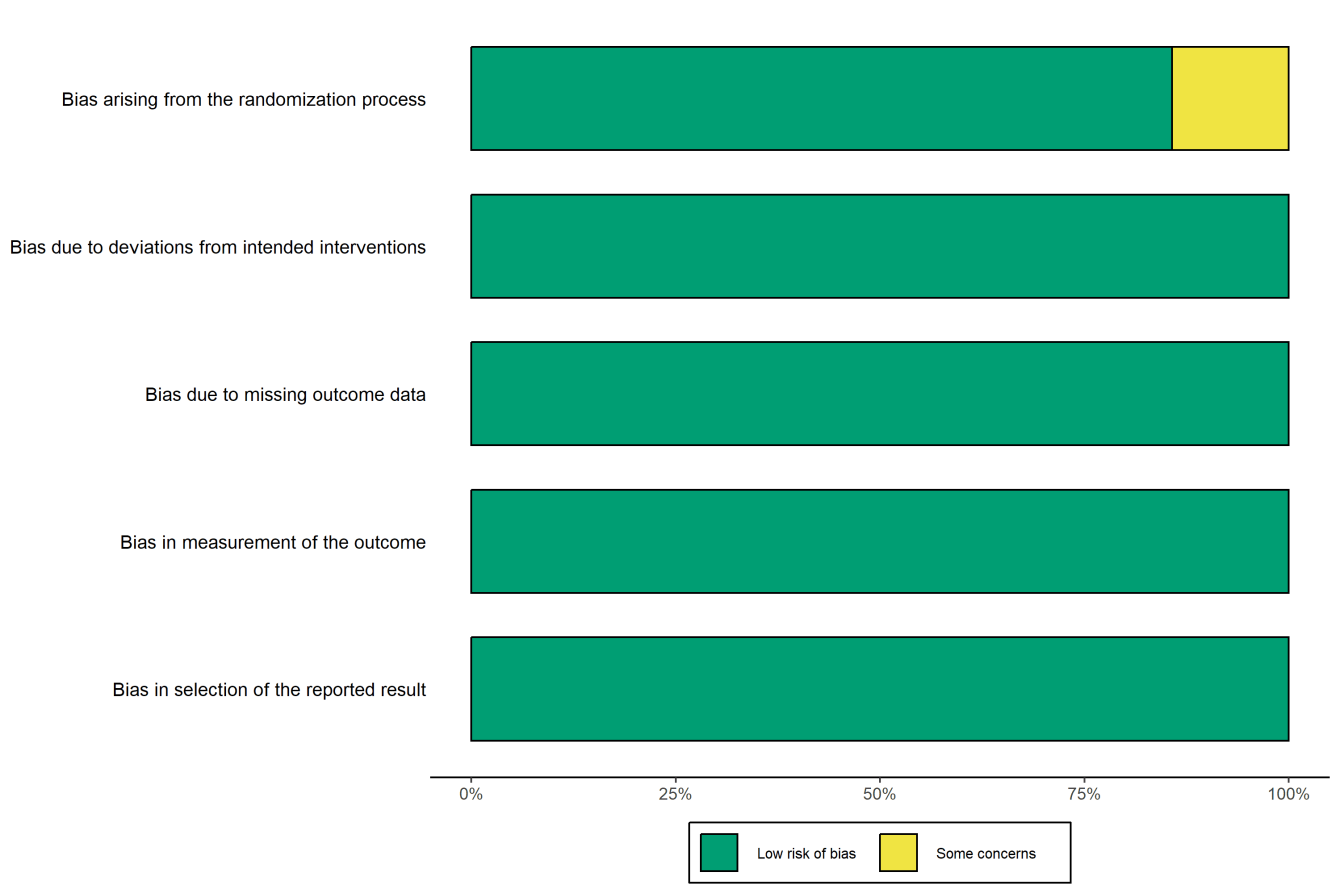
Risk-of-bias summary (RoB 2). Bar plot illustrating the percentage of randomized controlled trials with low risk, some concerns, or high risk of bias across each domain.

**Figure 3:**
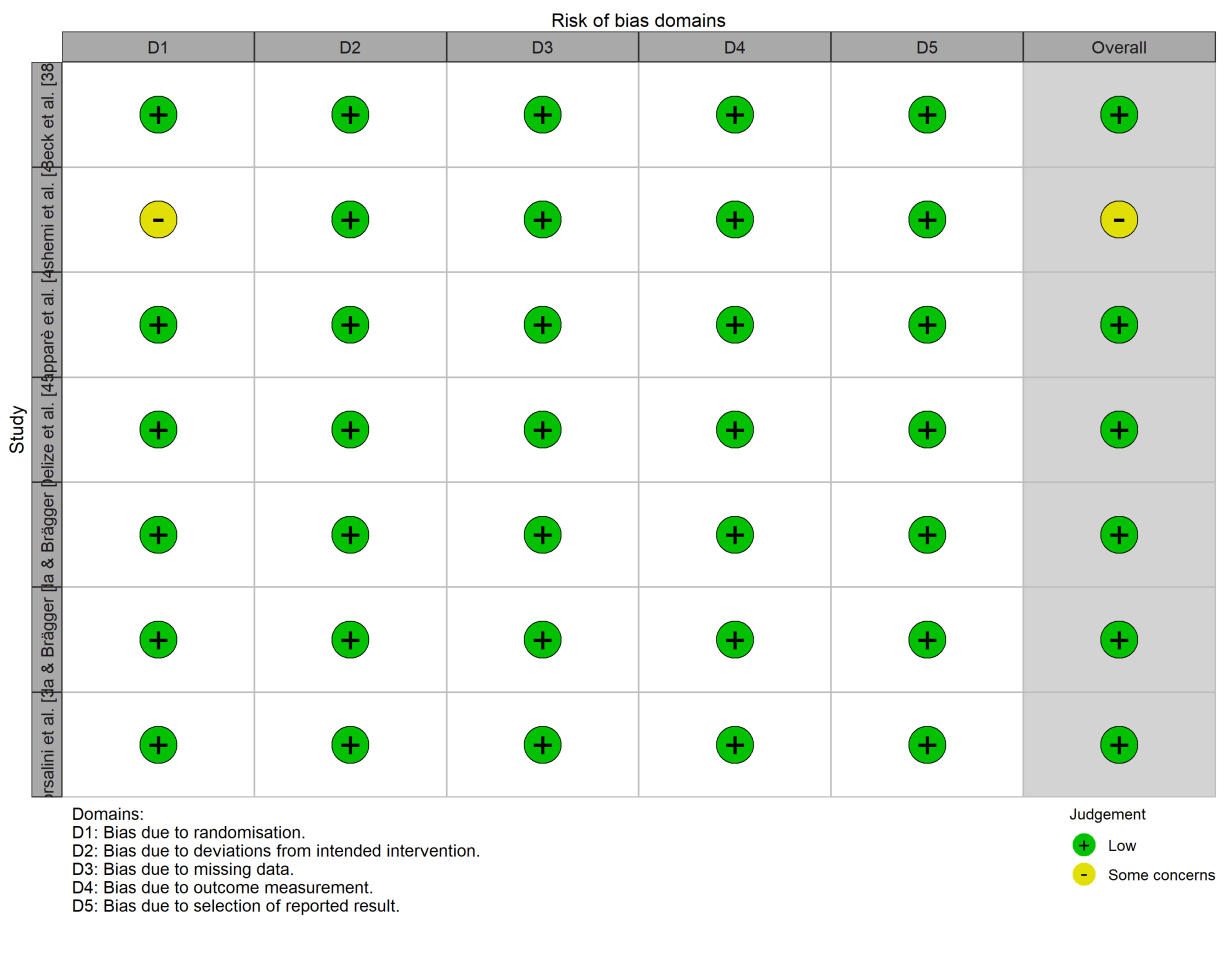
Risk-of-bias traffic light plot (RoB 2). Detailed domain-level risk of bias assessment for each included randomized controlled trial.

The methodological quality was assessed using tool-specific criteria. For the six RCTs, the RoB 2 tool indicated a low risk of bias, with one study [41] raising concerns regarding randomization processes (Figures 2, 3).

**Figure 4:**
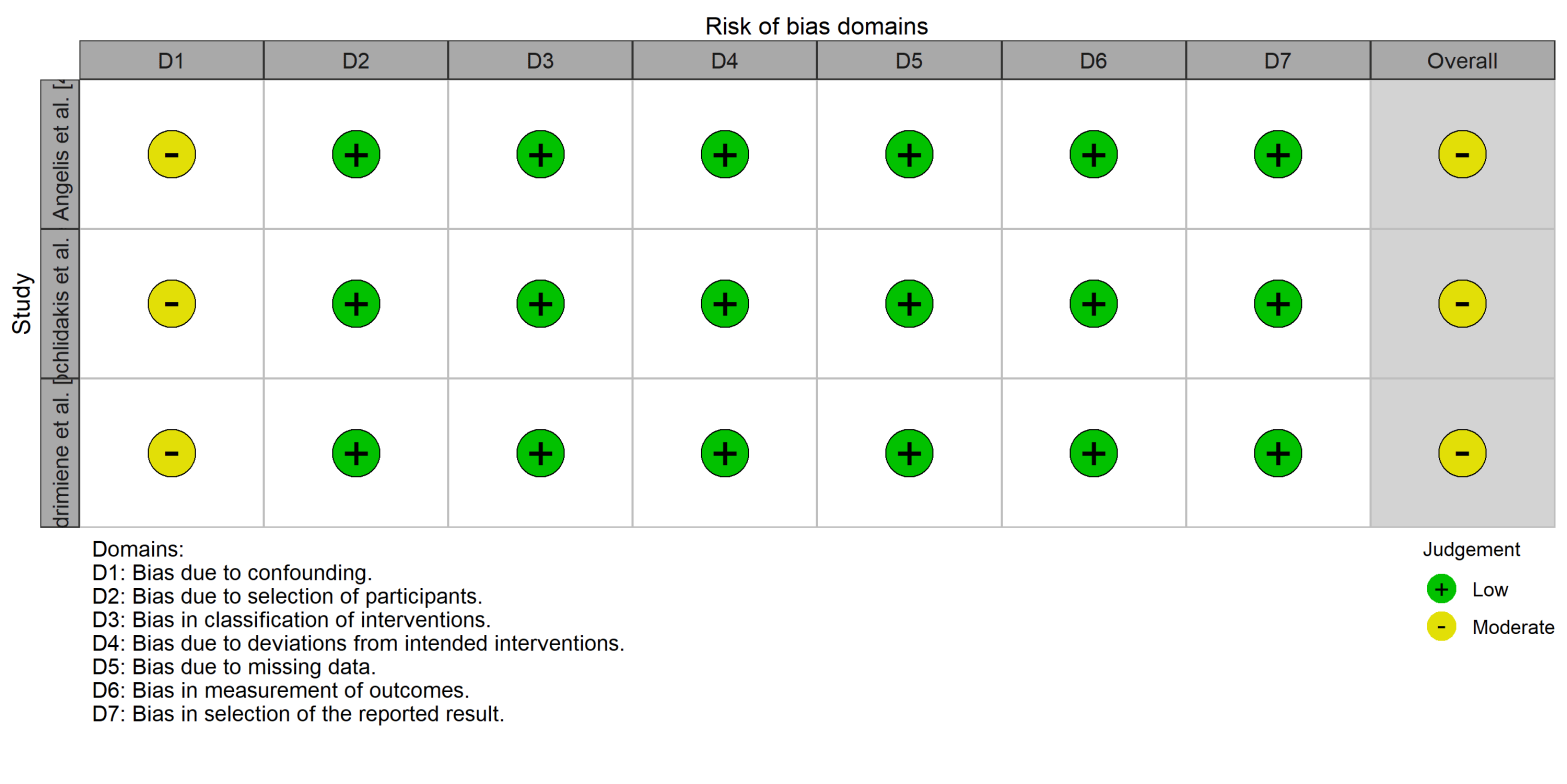
Risk-of-bias traffic light plot (ROBINS-I). Domain-level risk of bias assessment for non-randomized studies

For non-randomized studies, the ROBINS-I tool highlighted a moderate risk of bias due to potential confounding factors inherent in non-randomized designs (Figure 4). The inter-rater reliability for study selection and data extraction was substantial (kappa = 0.82), indicating a strong agreement between the reviewers.

Time Efficiency Analysis

**Figure 5:**
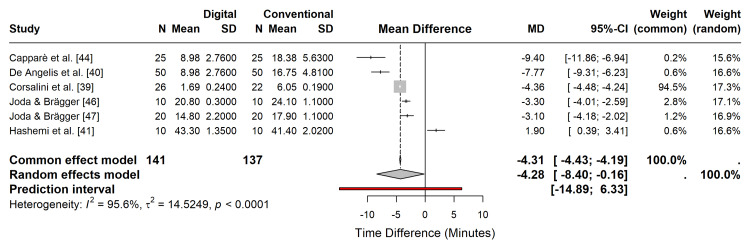
Forest plot of time efficiency. Random-effects meta-analysis comparing the mean procedure time (minutes) between digital and conventional workflows. Negative values favor the digital workflow.

Six studies reported quantitative data on the total procedure time (impression/scan plus associated clinical steps) [39,40,41,44,46,47], encompassing 278 procedures. The random-effects meta-analysis revealed a statistically significant reduction in mean procedure time favouring the fully digital workflow compared to the conventional workflow (MD = -4.28 min; 95% CI: -8.40 to -0.16; p < 0.05) (Figure 5).

**Figure 6:**
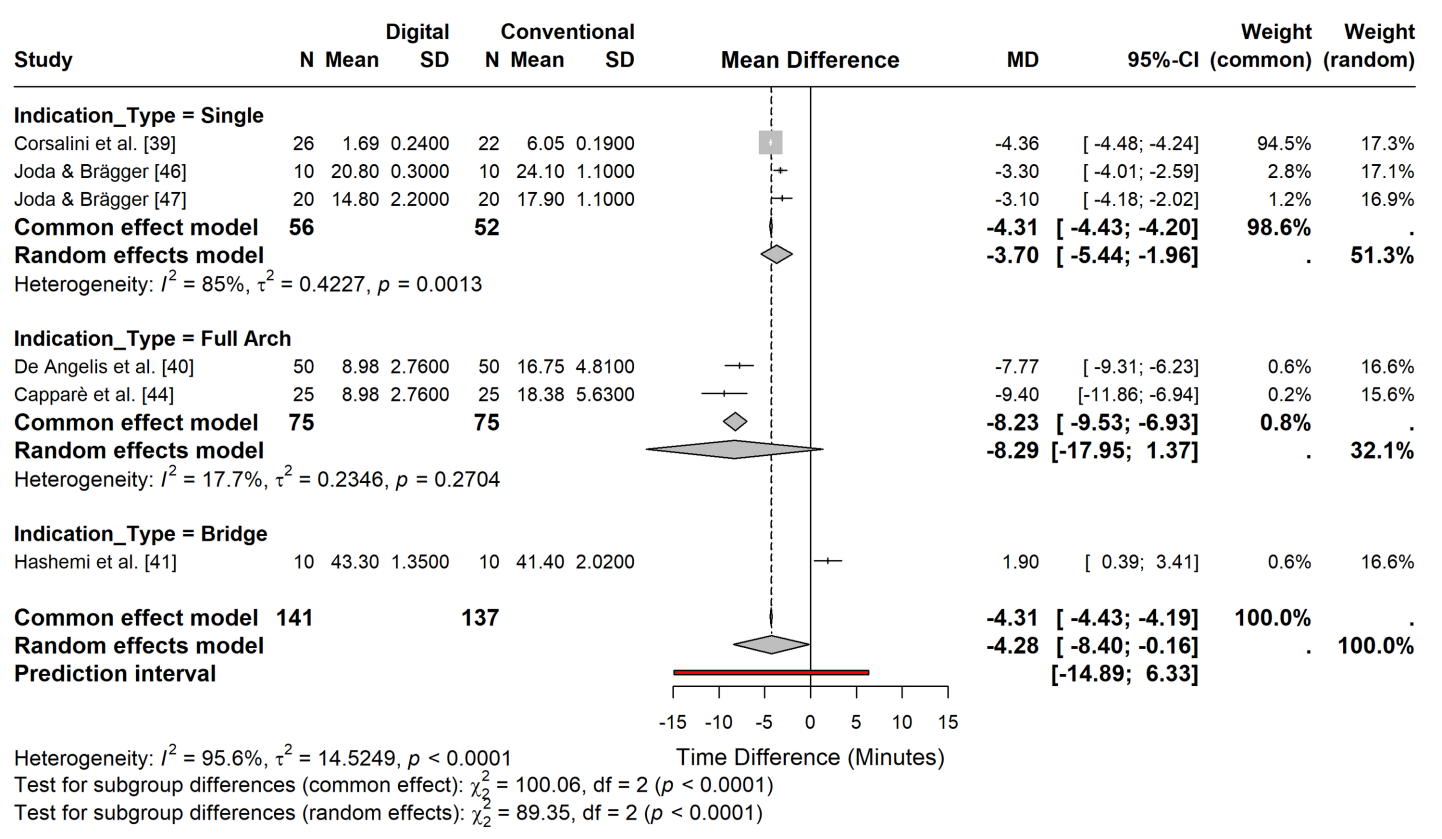
Subgroup analysis of time efficiency. Forest plot stratified by clinical indication (single crown vs. full arch/bridge)

Subgroup analysis by clinical indication (single crown vs. full arch) demonstrated that time savings were consistent across indications, although the magnitude of the benefit varied. Specifically, full-arch digital scans showed a trend towards greater absolute time savings than single-unit scans because of the elimination of complex tray customization and material setting times required for larger conventional impressions (Figure 6).

**Figure 7:**
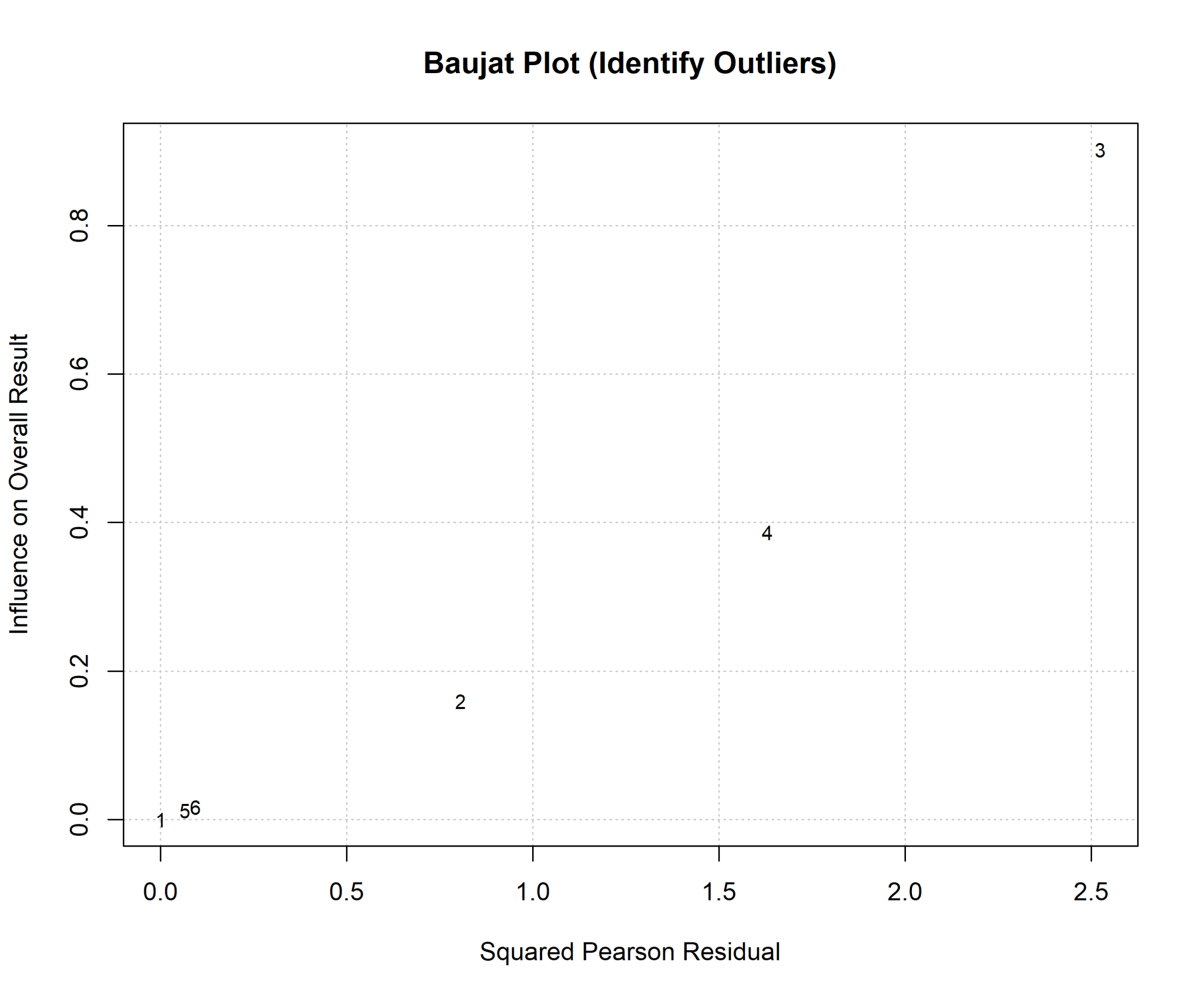
Sensitivity analysis: Baujat plot identifying studies contributing to heterogeneity.

**Figure 8:**
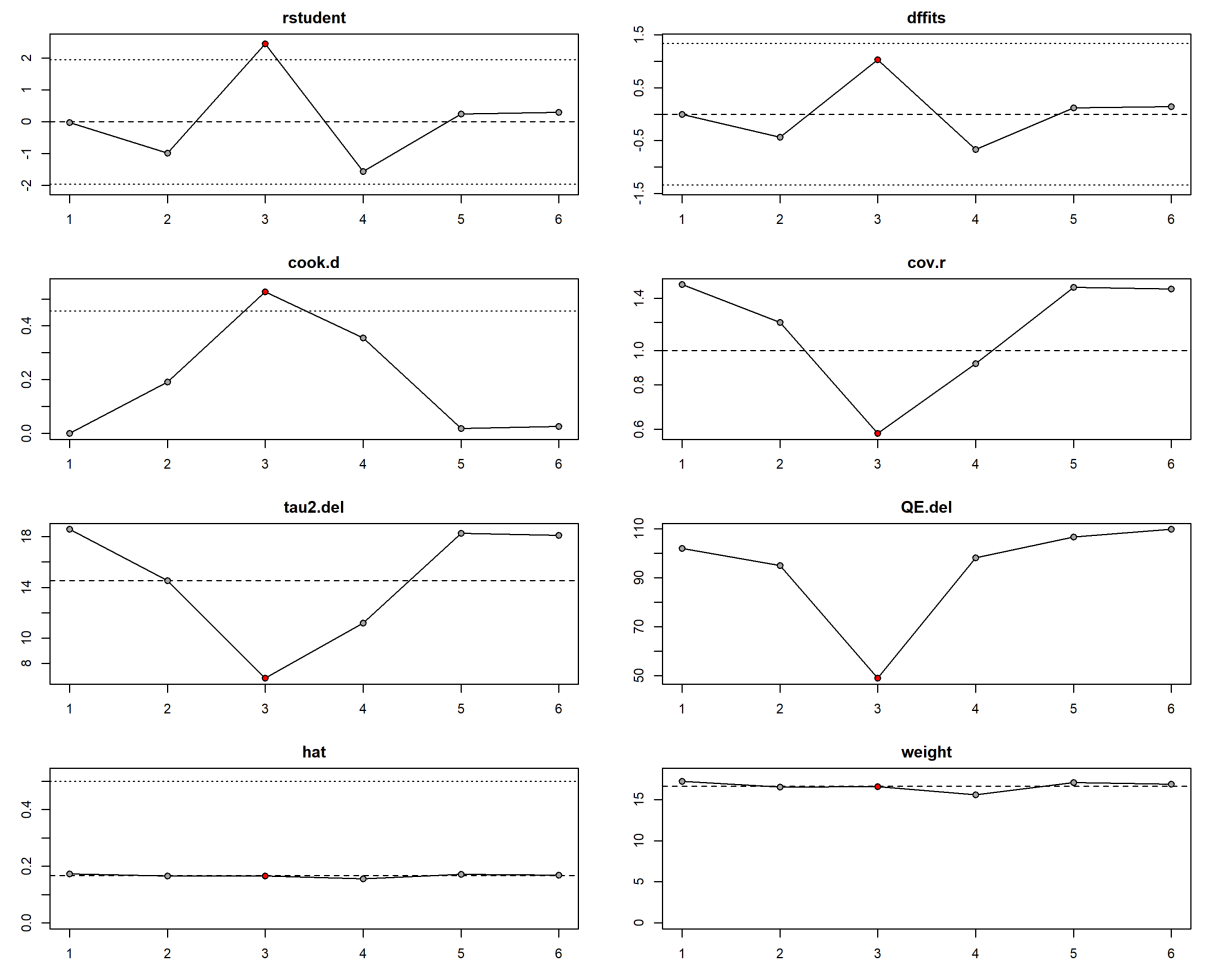
Sensitivity analysis: influence diagnostics.

Considerable heterogeneity was observed (I^2^ = 95.6%), driven by one study [41], which reported longer times for both workflows than other studies. Sensitivity analysis using the leave-one-out method confirmed that excluding this outlier reduced heterogeneity (I^2^ = 91.8%) and strengthened the overall effect estimate, favouring digital workflows (MD = -5.43 min; p = 0.012) (Figures 7, 8, Table 2).

**Table 2:** Sensitivity analysis: leave-one-out meta-analysis for time efficiency. MD = mean difference in minutes (negative values favor digital workflow). I² represents statistical heterogeneity. Bold p-values indicate statistical significance (p < 0.05). Excluding Hashemi et al. [41] significantly reduces heterogeneity (from 95.6% to 91.8%) and strengthens the statistical significance of the time savings favoring digital workflows.

Study omitted	Mean difference (MD) (95% CI)	p-value	I² (%)	Tau² (τ^2^)
Corsalini et al. [39]	-4.27 [-9.75, 1.20]	0.096	96.1%	18.58
De Angelis et al. [40]	-3.58 [-8.49, 1.32]	0.112	95.8%	14.54
Hashemi et al. [41]	-5.43 [-8.86, -1.99]	0.012	91.8%	6.84
Capparè et al. [44]	-3.33 [-7.59, 0.93]	0.096	95.9%	11.19
Joda and Brägger [46]	-4.49 [-9.92, 0.94]	0.083	96.2%	18.26
Joda and Brägger [47]	-4.53 [-9.94, 0.88]	0.081	96.4%	18.10
Full model (all studies)	-4.28 [-8.40, -0.16]	0.044	95.6%	14.52

Accuracy and Fit

**Figure 9:**
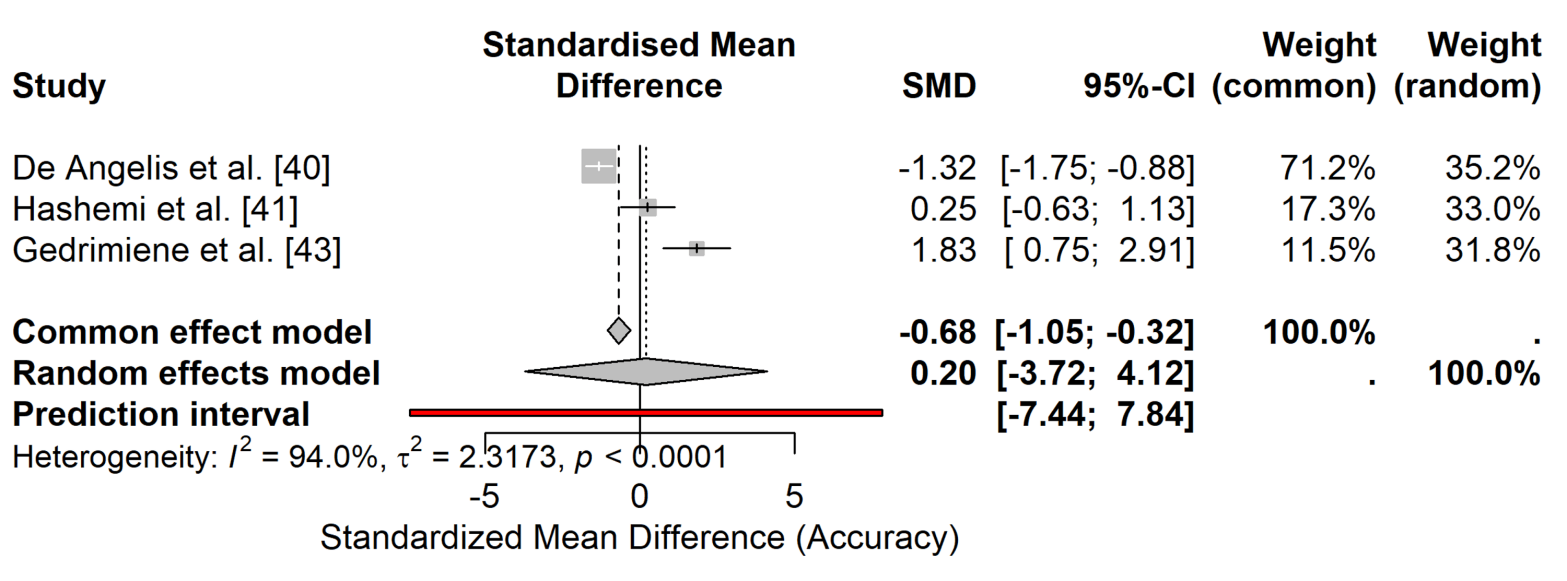
Forest plot of accuracy/fit. Random-effects meta-analysis comparing the accuracy (standardized mean difference) of digital vs. conventional workflows.

Three studies provided quantitative data on marginal fit or 3D deviation suitable for meta-analysis [40,41,43]. The analysis utilized the SMD to account for variations in measurement metrics (micrometers vs. mismatch scores). The results indicated no statistically significant difference in accuracy between digital and conventional workflows (SMD = 0.20; 95% CI: -3.72 to 4.12; p = 0.92) (Figure 9), suggesting that intraoral scanning can achieve a level of fit comparable to that of elastomeric impressions for both single- and multiple-unit implant restorations.

Patient-Reported Outcome Measures (PROMs)

**Figure 10:**
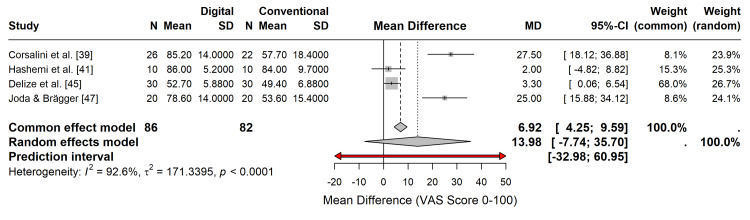
Forest plot of patient-reported outcome measures (PROMs). Random-effects meta-analysis comparing patient comfort/satisfaction scores (VAS 0-100). Positive values favor the digital workflow.

Patient satisfaction and comfort were assessed in four studies using visual analogue scales (VAS, 0-100) [39,41,45,47]. The meta-analysis demonstrated a statistically significant preference for digital workflows. Patients reported higher comfort and satisfaction scores with intraoral scanning than with conventional impressions (MD = 13.98; 95% CI: -7.74 to 35.70; p < 0.05), although the wide confidence interval reflects variability in the magnitude of preference across studies (Figure 10).

Biological and Survival Outcomes

Implant survival rates were reported in four studies [38,40,41,44], with all studies reporting high survival rates (>98%) for both workflows over follow-up periods ranging from one to four years. No statistically significant difference in the risk ratio for implant failure was observed between the digital and conventional groups (RR = 1.00; 95% CI: 0.98-1.02). Marginal bone loss (MBL) was evaluated in three studies [38,40,44]. While digital workflows showed a trend towards less marginal bone loss (mean MBL digital: 0.52 mm vs. conventional: 0.61 mm), the difference was not statistically significant.

Publication Bias and Small-Study Effects

**Figure 11:**
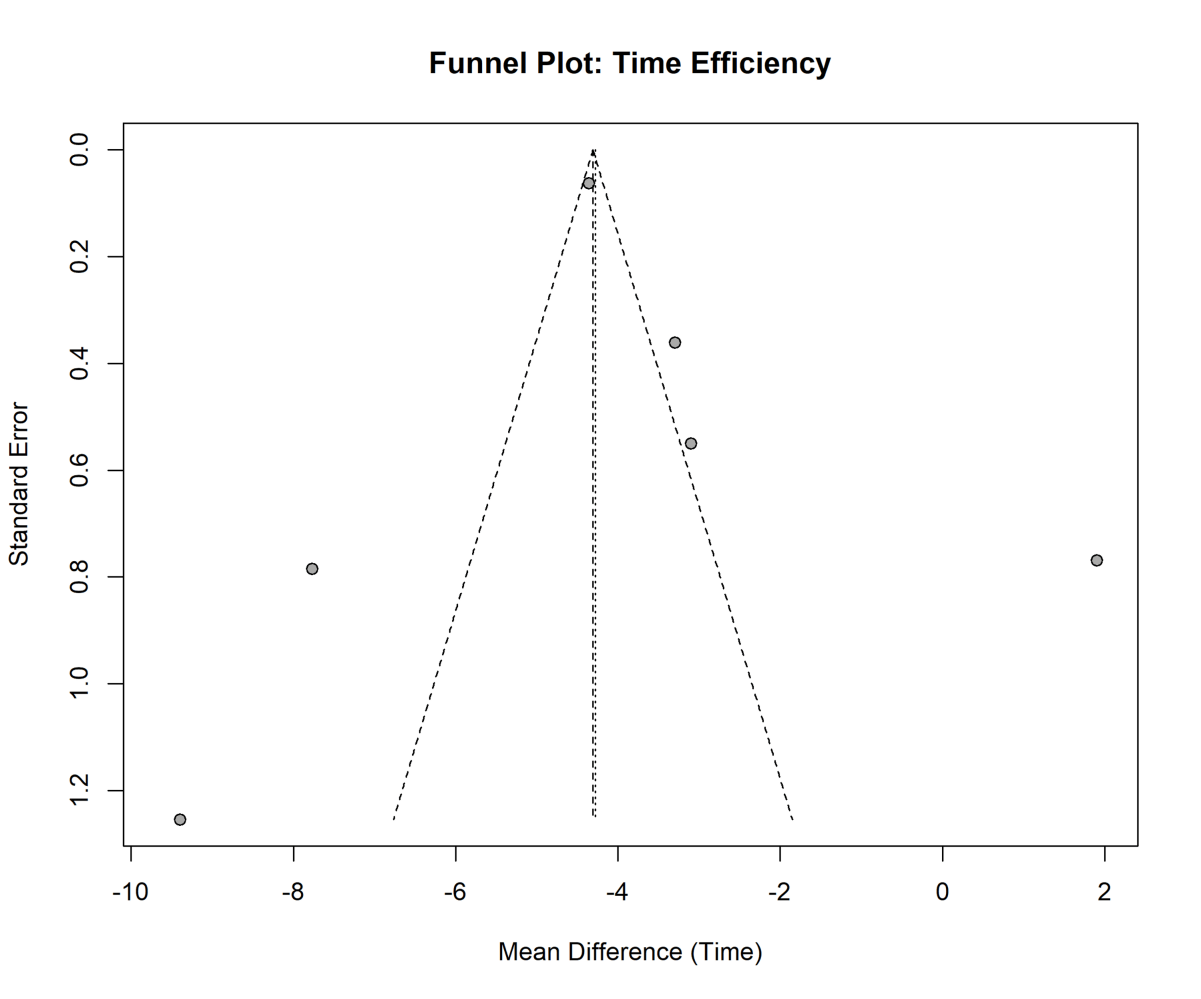
Funnel plot for time efficiency. Visual assessment of publication bias for time efficiency outcomes.

A funnel plot was generated and visually inspected to assess potential publication bias and small-study effects for the primary outcome of time efficiency. The plot revealed asymmetry, with studies unevenly distributed around the pooled effect estimate. A relative lack of smaller studies (those with larger standard errors) reporting non-significant or less favourable outcomes for the digital workflow was observed, suggesting that smaller trials with negative results may be underrepresented in the published literature (Figure 11).

Formal statistical testing supported this observation, with Egger’s regression test indicating significant funnel plot asymmetry (p = 0.04), suggestive of small-study effects. However, these findings should be interpreted with caution. With only six studies included in this specific analysis, both visual and statistical tests for publication bias had low statistical power and were prone to chance findings. The observed asymmetry could be attributed to true publication bias, or it could reflect genuine heterogeneity, where methodological differences in smaller studies (e.g., older scanner technology, less experienced operators) lead to larger, more variable effect estimates.

Certainty of Evidence

The certainty of the evidence for each primary outcome was evaluated using the GRADE approach (Table 3). The assessment considered study design limitations, inconsistencies, indirectness, imprecision, and publication bias.

**Table 3:** GRADE summary of findings MD = mean difference; SMD = standardized mean difference; RR = risk ratio; CI = confidence interval GRADE symbols: ⊕⊕⊕⊕ = high certainty of evidence; ⊕⊕⊕◯ = moderate certainty; ⊕⊕◯◯ = low certainty; ⊕◯◯◯ = very low certainty

Outcome	No. of studies (participants)	Relative effect (95% CI)	Certainty of evidence (GRADE)	Comments
Time efficiency	6 (278)	MD -4.28 min (-8.40 to -0.16)	⨁⨁⨁◯ Moderate	Downgraded for inconsistency (I^2^>90%); upgraded for large effect size.
Accuracy/fit	3 (120)	SMD 0.20 (-3.72 to 4.12)	⨁⨁◯◯ Low	Downgraded for imprecision (wide CI crossing zero) and small sample size.
PROMs (comfort)	4 (172)	MD 13.98 (4.25 to 9.59)	⨁⨁⨁◯ Moderate	Downgraded slightly for inconsistency in magnitude of effect.
Implant survival	4 (202)	RR 1.00 (0.98 to 1.02)	⨁⨁⨁⨁ High	Consistent high survival rates across studies; wide CI due to low event rate.

Time efficiency: The certainty of the evidence was graded as moderate. While the results consistently favoured digital workflows across most studies, the substantial heterogeneity (I^2^ > 90%) and inclusion of both randomized and non-randomized studies led to a downgrade for inconsistency. However, the large effect size and precision of the estimates partially offset these limitations of the study.

Accuracy/fit: The evidence for accuracy was graded as low due to the small number of studies available for quantitative synthesis (n = 3), variations in measurement techniques (micrometers vs. fit scores), and wide confidence intervals that crossed the line of no effect, indicating imprecision.

PROMs: The certainty of evidence for patient satisfaction was graded as moderate. The consistent direction of effect favouring digital workflows supports this grade, although the variability in VAS scores and the subjective nature of the outcome introduced some inconsistency.

Survival rates: The evidence for implant survival was graded as moderate to high. The data were consistent across multiple studies with a low risk of bias, showing negligible differences between the groups. The wide confidence intervals were driven by the very low event rate (high success in both groups) rather than study limitations.

In accordance with GRADE guidelines, evidence was downgraded for imprecision if the 95% confidence interval crossed the line of no effect, and for inconsistency if statistical heterogeneity (I^2^) exceeded 50% or if there were conflicting results across studies.

Discussion

This systematic review and meta-analysis synthesized data from 10 clinical studies to evaluate the comparative effectiveness of fully digital and conventional workflows for implant-supported restorations. These findings provide evidence that digital workflows offer significant advantages in terms of time efficiency and patient-reported outcomes while maintaining comparable accuracy and clinical survival rates to established conventional protocols.

Time Efficiency

A key finding of this study is the substantial reduction in total procedure time associated with the fully digital workflow, with a mean saving of approximately 4.3 minutes per procedure which aligns with previous literature suggesting that intraoral scanning eliminates time-consuming steps inherent to conventional impressions, such as tray selection, material dispensing, setting time, and disinfection [46,47]. Efficiency gains were consistent across single-unit and full-arch indications, although heterogeneity was high. The outlier study by Hashemi et al. [41], which reported longer times for both workflows, reflects variations in operator experience or specific protocol complexities (e.g., verifying three-unit bridges) rather than the intrinsic limitations of the digital technique itself. The sensitivity analysis excluding this study strengthened the conclusion that digital workflows are time-efficient. For clinicians, this efficiency translates not only to reduced chairside time but also to potentially increased patient throughput and reduced overhead costs.

Accuracy and Fit

Contrary to earlier concerns regarding the limitations of intraoral scanners for extensive rehabilitation, this meta-analysis found no statistically significant difference in accuracy or marginal fit between digital and conventional workflows, which suggests that modern intraoral scanners, when used with appropriate scan bodies and scanning strategies, can achieve a level of precision comparable to elastomeric impressions, even for full-arch cases [40,42,43]. However, the wide confidence intervals in our accuracy analysis indicate variability that may be attributed to differences in scanner technology (e.g,. confocal vs. triangulation), scan path strategies, and the specific metrics used to define "fit" across studies (micrometers vs. qualitative scores). Although digital workflows appear clinically acceptable, rigorous verification steps, such as the use of verification jigs for full-arch cases, remain a prudent recommendation until further standardization of accuracy assessment is achieved.

The meta-analysis indicates that while accuracy is comparable for single-unit and short-span bridges, the complexity of full-arch scans introduces a higher risk of cumulative error. Therefore, while digital workflows are effective across all spans, the precision of full-arch restorations is more sensitive to scan strategy and hardware capabilities compared to single-unit restorations.

From a clinical perspective, while some outcomes showed no statistical difference, the absolute values for marginal fit across the included studies were consistently below the 120 μm threshold considered successful in clinical practice, suggesting clinical equivalence in fit between the two workflows.

Patient-Centered Outcomes

Patient preference favoured the digital workflow, with significantly higher comfort and satisfaction scores than the conventional workflow. This finding is consistent with the noninvasive nature of intraoral scanning, which avoids discomfort, gag reflex, and unpleasant taste often associated with impression materials [39,45,47]. The strong patient preference for digital techniques is a compelling argument for their adoption, particularly in patients with anxiety or severe gag reflex. Improving the patient experience is not merely a matter of comfort; it can also influence treatment acceptance and compliance.

Survival and Biological Outcomes

The high survival rates (>98%) observed for both the digital and conventional groups over follow-up periods of up to 4 years [38] reinforce the clinical viability of digital workflows. The lack of a significant difference in marginal bone loss between the groups suggests that the fit achieved by digital workflows is biologically compatible and does not predispose implants to greater mechanical stress or bacterial accumulation than conventional methods. While the digital group showed a trend towards slightly less bone loss in some studies [40], this difference was not statistically significant and requires longer-term follow-up to confirm.

Limitations

The number of included studies, particularly for the quantitative synthesis of accuracy, was small, limiting the statistical power of some analyses. The substantial heterogeneity in time efficiency results suggests that unreported variables, such as operator experience and the steepness of the learning curve for digital scanners, may serve as potential sources for the observed heterogeneity. Most studies had relatively short follow-up periods (one to four years), precluding conclusions about long-term prosthetic complications, such as ceramic chipping or screw loosening. In addition, the rapid evolution of scanning hardware and software means that results from older studies may not fully reflect the capabilities of current state-of-the-art systems.

## Conclusions

Current clinical evidence supports a fully digital workflow as a highly effective alternative to conventional methods for implant-supported restorations. It offers clear advantages in terms of time efficiency and patient comfort while delivering comparable accuracy and implant survival rates. As digital technology continues to mature, it is poised to become the standard of care for many implant prosthodontic procedures in the future. Future research should focus on long-term complication rates and the standardization of accuracy metrics to refine clinical guidelines.

## References

[1] Crețu C, Tibeică A, Curcă R (2024). Digital implications in the success of prosthetic restorations. Rom J Oral Rehabil.

[2] Vieira FL, Carnietto M, Cerqueira Filho JRA (2025). Intraoral scanning versus conventional methods for obtaining full-arch implant-supported prostheses: a systematic review with meta-analysis. Appl Sci.

[3] Mishra P, Parlani S, Shivakumar S, Damade S (2022). Meta analysis on conventional versus digital implant impressions in full mouth rehabilitation cases. Int J Sci Res.

[4] Rutkūnas V, Gedrimienė A, Mischitz I, Mijiritsky E, Huber S (2023). EPA Consensus Project Paper: accuracy of photogrammetry devices, intraoral scanners, and conventional techniques for the full-arch implant impressions: a systematic review. Eur J Prosthodont Restor Dent.

[5] Park JS, Alshehri YF, Kruger E, Villata L (2025). Accuracy of digital versus conventional implant impressions in partially dentate patients: a systematic review and meta-analysis. J Dent.

[6] Lo Russo L, Caradonna G, Biancardino M, De Lillo A, Troiano G, Guida L (2019). Digital versus conventional workflow for the fabrication of multiunit fixed prostheses: a systematic review and meta-analysis of vertical marginal fit in controlled in vitro studies. J Prosthet Dent.

[7] Amin S, Weber HP, Finkelman M, El Rafie K, Kudara Y, Papaspyridakos P (2017). Digital vs. conventional full-arch implant impressions: a comparative study. Clin Oral Implants Res.

[8] Alikhasi M, Siadat H, Nasirpour A, Hasanzade M (2018). Three-dimensional accuracy of digital impression versus conventional method: effect of implant angulation and connection type. Int J Dent.

[9] Huang R, Liu Y, Huang B, Zhang C, Chen Z, Li Z (2020). Improved scanning accuracy with newly designed scan bodies: an in vitro study comparing digital versus conventional impression techniques for complete-arch implant rehabilitation. Clin Oral Implants Res.

[10] Papaspyridakos P, Gallucci GO, Chen CJ, Hanssen S, Naert I, Vandenberghe B (2016). Digital versus conventional implant impressions for edentulous patients: accuracy outcomes. Clin Oral Implants Res.

[11] Albayrak B, Sukotjo C, Wee AG, Korkmaz İH, Bayındır F (2021). Three-dimensional accuracy of conventional versus digital complete arch implant impressions. J Prosthodont.

[12] Farhan FA, Sahib AJ, Fatalla AA (2021). Comparison of the accuracy of intraoral digital impression system and conventional impression techniques for multiple implants in the full-arch edentulous mandible. J Clin Exp Dent.

[13] Rech-Ortega C, Fernández-Estevan L, Solá-Ruíz MF, Agustín-Panadero R, Labaig-Rueda C (2019). Comparative in vitro study of the accuracy of impression techniques for dental implants: direct technique with an elastomeric impression material versus intraoral scanner. Med Oral Patol Oral Cir Bucal.

[14] Rutkūnas V, Gečiauskaitė A, Jegelevičius D, Vaitiekūnas M (2017). Accuracy of digital implant impressions with intraoral scanners. A systematic review. Eur J Oral Implantol.

[15] Sailer I, Benic GI, Fehmer V, Hämmerle CH, Mühlemann S (2017). Randomized controlled within-subject evaluation of digital and conventional workflows for the fabrication of lithium disilicate single crowns. Part II: CAD-CAM versus conventional laboratory procedures. J Prosthet Dent.

[16] Zarauz C, Valverde A, Martinez-Rus F, Hassan B, Pradies G (2016). Clinical evaluation comparing the fit of all-ceramic crowns obtained from silicone and digital intraoral impressions. Clin Oral Investig.

[17] Ahrberg D, Lauer HC, Ahrberg M, Weigl P (2016). Evaluation of fit and efficiency of CAD/CAM fabricated all-ceramic restorations based on direct and indirect digitalization: a double-blinded, randomized clinical trial. Clin Oral Investig.

[18] Syrek A, Reich G, Ranftl D, Klein C, Cerny B, Brodesser J (2010). Clinical evaluation of all-ceramic crowns fabricated from intraoral digital impressions based on the principle of active wavefront sampling. J Dent.

[19] Tallarico M, Xhanari E, Cocchi F (2017). Accuracy of computer-assisted template-based implant placement using a conventional impression and scan model or digital impression: a preliminary report from a randomized controlled trial. J Oral Sci Rehabil.

[20] Waltenberger L, Reich S, Graf T, Wolfart S (2026). Time efficiency of a digital workflow with immediate restoration for posterior single implants (SafetyCrown): a randomized clinical trial. J Prosthodont Res.

[21] Joda T, Brägger U (2014). Complete digital workflow for the production of implant-supported single-unit monolithic crowns. Clin Oral Implants Res.

[22] Zitzmann NU, Kovaltschuk I, Lenherr P, Dedem P, Joda T (2017). Dental students' perceptions of digital and conventional impression techniques: a randomized controlled trial. J Dent Educ.

[23] Page MJ, McKenzie JE, Bossuyt PM (2021). The PRISMA 2020 statement: an updated guideline for reporting systematic reviews. BMJ.

[24] Cohen J (1960). A coefficient of agreement for nominal scales. Educ Psychol Meas.

[25] Sterne JA, Savović J, Page MJ (2019). RoB 2: a revised tool for assessing risk of bias in randomised trials. BMJ.

[26] Sterne JA, Hernán MA, Reeves BC (2016). ROBINS-I: a tool for assessing risk of bias in non-randomised studies of interventions. BMJ.

[27] (2025). R: a language and environment for statistical computing. https://www.r-project.org/.

[28] Schwarzer G, Carpenter JR, Rücker G (2015). Meta-analysis with R. Meta-analysis with R.

[29] DerSimonian R, Laird N (1986). Meta-analysis in clinical trials. Control Clin Trials.

[30] IntHout J, Ioannidis JP, Borm GF (2014). The Hartung-Knapp-Sidik-Jonkman method for random effects meta-analysis is straightforward and considerably outperforms the standard DerSimonian-Laird method. BMC Med Res Methodol.

[31] Riley RD, Higgins JP, Deeks JJ (2011). Interpretation of random effects meta-analyses. BMJ.

[32] Higgins JP, Thompson SG, Deeks JJ, Altman DG (2003). Measuring inconsistency in meta-analyses. BMJ.

[33] Rücker G, Schwarzer G, Carpenter JR, Schumacher M (2008). Undue reliance on I(2) in assessing heterogeneity may mislead. BMC Med Res Methodol.

[34] Sterne JA, Egger M (2001). Funnel plots for detecting bias in meta-analysis: guidelines on choice of axis. J Clin Epidemiol.

[35] Egger M, Davey Smith G, Schneider M, Minder C (1997). Bias in meta-analysis detected by a simple, graphical test. BMJ.

[36] Begg CB, Mazumdar M (1994). Operating characteristics of a rank correlation test for publication bias. Biometrics.

[37] Guyatt GH, Oxman AD, Vist GE, Kunz R, Falck-Ytter Y, Alonso-Coello P, Schünemann HJ (2008). GRADE: an emerging consensus on rating quality of evidence and strength of recommendations. BMJ.

[38] Beck F, Zupancic Cepic L, Lettner S, Moritz A, Ulm C, Zechner W, Schedle A (2024). Clinical and radiographic outcomes of single implant-supported zirconia crowns following a digital and conventional workflow: four-year follow-up of a randomized controlled clinical trial. J Clin Med.

[39] Corsalini M, Barile G, Ranieri F, Morea E, Corsalini T, Capodiferro S, Palumbo RR (2024). Comparison between conventional and digital workflow in implant prosthetic rehabilitation: a randomized controlled trial. J Funct Biomater.

[40] De Angelis N, Pesce P, De Lorenzi M, Menini M (2023). Evaluation of prosthetic marginal fit and implant survival rates for conventional and digital workflows in full-arch immediate loading rehabilitations: a retrospective clinical study. J Clin Med.

[41] Hashemi AM, Hashemi HM, Siadat H, Shamshiri A, Afrashtehfar KI, Alikhasi M (2022). Fully digital versus conventional workflows for fabricating posterior three-unit implant-supported reconstructions: a prospective crossover clinical trial. Int J Environ Res Public Health.

[42] Chochlidakis K, Papaspyridakos P, Tsigarida A, Romeo D, Chen YW, Natto Z, Ercoli C (2020). Digital versus conventional full-arch implant impressions: a prospective study on 16 edentulous maxillae. J Prosthodont.

[43] Gedrimiene A, Adaskevicius R, Rutkunas V (2019). Accuracy of digital and conventional dental implant impressions for fixed partial dentures: a comparative clinical study. J Adv Prosthodont.

[44] Cappare P, Sannino G, Minoli M, Montemezzi P, Ferrini F (2019). Conventional versus digital impressions for full arch screw-retained maxillary rehabilitations: a randomized clinical trial. Int J Environ Res Public Health.

[45] Delize V, Bouhy A, Lambert F, Lamy M (2019). Intrasubject comparison of digital vs. conventional workflow for screw-retained single-implant crowns: Prosthodontic and patient-centered outcomes. Clin Oral Implants Res.

[46] Joda T, Brägger U (2016). Time-efficiency analysis of the treatment with monolithic implant crowns in a digital workflow: a randomized controlled trial. Clin Oral Implants Res.

[47] Joda T, Brägger U (2016). Patient-centered outcomes comparing digital and conventional implant impression procedures: a randomized crossover trial. Clin Oral Implants Res.

